# Development of an Innovative Digital Data Collection System for Routine Mental Health Care Delivery in Rural Haiti

**DOI:** 10.9745/GHSP-D-20-00486

**Published:** 2021-12-31

**Authors:** Alexandra L. Rose, Darius L. Fenelon, J. Reginald Fils-Aimé, Wilder Dubuisson, Sarah F.C. Singer, Stephanie L. Smith, Gregory Jerome, Eddy Eustache, Giuseppe Raviola

**Affiliations:** a Department of Psychology, University of Maryland, College Park, MD, USA.; b Partners In Health, Boston, MA, USA.; c Zanmi Lasante, Croix-des-Bouquets, Haiti.; d Department of Global Health and Social Medicine, Harvard Medical School, Boston, MA, USA.; e Brigham and Women's Hospital, Boston, MA, USA.

## Abstract

**Introduction:**

Effective digital health management information systems (HMIS) support health data validity, which enables health care teams to make programmatic decisions and country-level decision making in support of international development targets. In 2015, mental health was included within the Sustainable Development Goals, yet there are few applications of HMIS of any type in the practice of mental health care in resource-limited settings. Zanmi Lasante (ZL), one of the largest providers of mental health care in Haiti, developed a digital data collection system for mental health across 11 public rural health facilities.

**Program Intervention:**

We describe the development, implementation, and evaluation of the digital system for mental health data collection at ZL. To evaluate system reliability, we assessed the number of missing monthly reports. To evaluate data validity, we calculated concordance between the digital system and paper charts at 2 facilities. To evaluate the system's ability to inform decision making, we specified and then calculated 4 priority indicators.

**Results:**

The digital system was missing 5 of 143 monthly reports across all facilities and had 74.3% (55/74) and 98% (49/50) concordance with paper charts. It was possible to calculate all 4 indicators, which led to programmatic changes in 2 cases. In response to implementation challenges, it was necessary to use strategies to increase provider buy-in and ultimately to introduce dedicated data clerks to keep pace with data collection and protect time for clinical work.

**Lessons Learned:**

While demonstrating the potential of collecting mental health data digitally in a low-resource rural setting, we found that it was necessary to consider the ongoing roles of paper records alongside digital data collection. We also identified the challenge of balancing clinical and data collection responsibilities among a limited staff. Ongoing work is needed to develop truly sustainable and scalable models for mental health data collection in resource-limited settings.

## Abstract (precis)

Mental health information systems in low-resource settings are scarce worldwide. Data collection was accurate, yet sustainable staffing was a challenge when using task-shared clinical providers for data collection in health centers in rural Haiti. Integrating mental health data collection within existing data collection systems would help close this key gap.

## Teaser

**Key Findings:**

- Balancing time for clinical duties with time for data collection was a key challenge when using task-shared health care workers to collect data.
- Feedback of data to users, ongoing supervision, and opportunities for rewards for high-performers may help increase provider buy-in for new data collection systems.
- Retaining paper forms alongside digital data collection systems or the inclusion of decision support tools directly within digital data collection systems may be needed to fully support health care worker learning in a task-shared mental health system.

**Key Implications:**

- Program managers should carefully consider sustainable staffing from the beginning when designing digital data collection projects.
- Program managers should consider how digital data collection systems can incorporate decision support tools to sustainably support ongoing learning of task-shared providers.
- Policy makers should integrate mental health data collection within other data systems to leverage infrastructure and resources for mental health.

Résumé en français à la fin de l'article.

## INTRODUCTION

In large and complex health care settings, electronic health management information systems (HMIS) play a crucial role in ensuring that high-quality and valid data are routinely collected.^1^^–^^3^ The ability to make decisions informed by data is essential for providers to be able to provide high-quality longitudinal care and for countries to be able to track services, measure the quality of care delivered, and work toward international development targets, such as the Sustainable Development Goals (SDGs). For the first time in 2015, the SDGs included mental health, bolstering the emerging movement to expand access to quality mental health care globally.^4^^,^^5^ However, in resource-limited settings, there are few examples of the application of electronic HMIS in mental health care delivery, making evaluation and tracking of services challenging.^6^^–^^9^ In Haiti, as in many other low- and middle-income countries (LMICs), there is no national HMIS for mental health.^10^

There is a need for the development, implementation, and evaluation of digital data collection systems for mental health, particularly those that can be deployed in resource-limited settings.^11^ Nongovernmental organizations (NGOs) involved in developing and delivering mental health services in low-resource contexts where mental health data collection is limited or nonexistent can contribute by helping develop and test mental health electronic data collection systems within their programs.^12^ This article describes a digital data collection system for mental health data developed, implemented, and evaluated in rural Haiti. We identify key lessons learned about digital mental health data collection in low-resource settings.

## PROJECT CONTEXT

**FIGURE 1:**
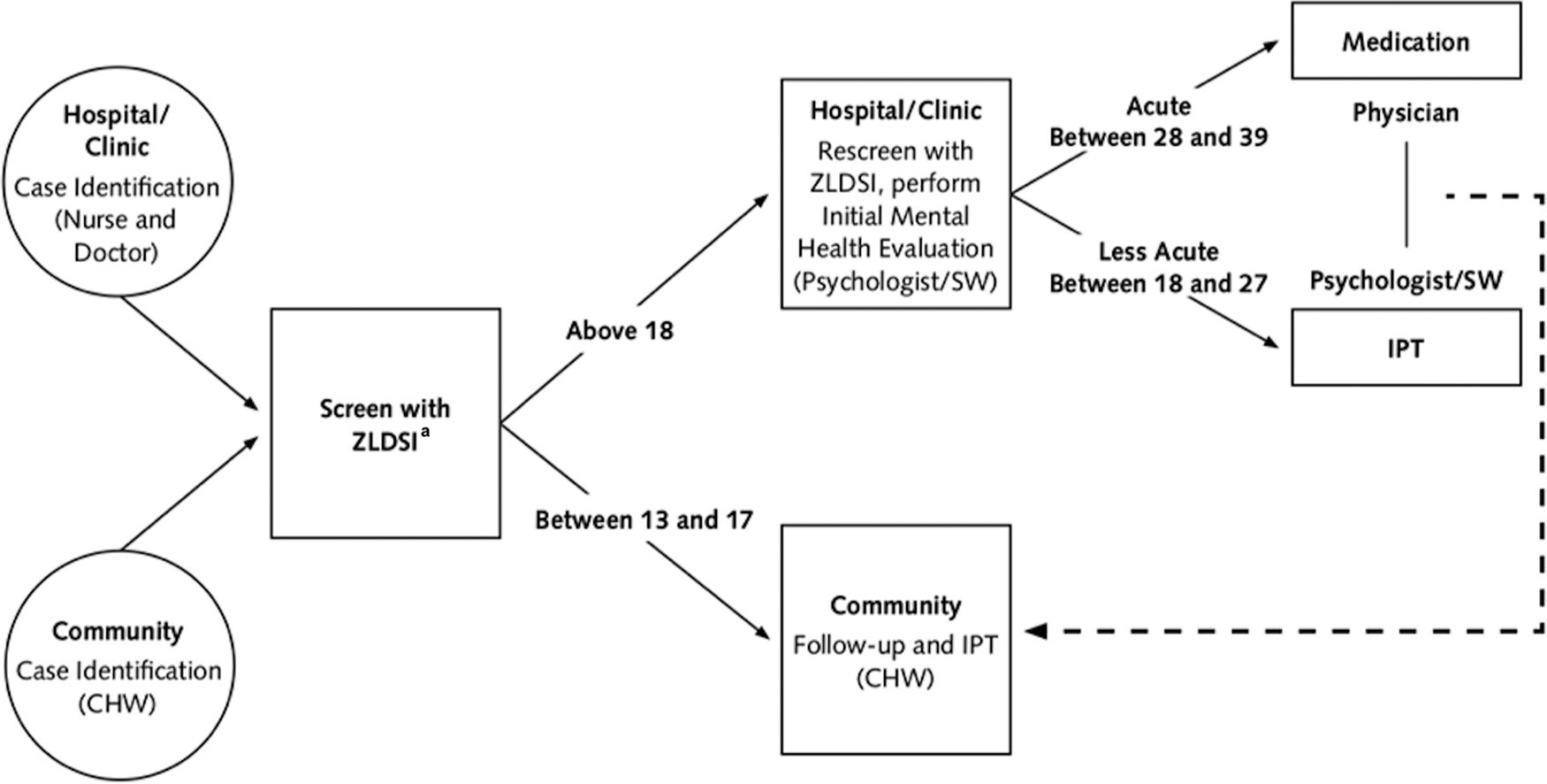
Zanmi Lasante Depression Care Pathway Used at Health Facilities in Rural Haiti Abbreviations: CHW, community health worker; IPT, interpersonal psychotherapy; SW, social worker; ZLDSI, Zanmi Lasante Depression Symptom Inventory. ^a^Administer the Zanmi Lasante Depression Symptom Inventory every 2 weeks. Illustration prepared by Partners in Health.

Zanmi Lasante (ZL) is a partner organization of the health systems strengthening nonprofit organization Partners In Health (PIH). ZL has provided health care in rural Haiti for more than 30 years and serves a primary catchment area of more than 1.3 million people across 11 rural public health facilities in the Central Plateau and Lower Artibonite departments through a public-private partnership with the Haiti Ministry of Health. After the 2010 earthquake, ZL developed and implemented a mental health system using the “5 x 5” framework, which defines 5 minimum skills packages needed to provide basic mental health care and 5 implementation rules to support the implementation of task-shared mental health care in a low-resource health system.^13^ The ZL mental health care system is described in detail elsewhere.^14^^–^^16^ In brief, the system of mental health care provided at ZL uses a task-sharing model, in which nonspecialists, including bachelor's level psychologists, who may have had limited clinical practice when hired at ZL, and community health workers, deliver components of care.^17^ Persons who screen positive for a possible mental health diagnosis in either the community or at a facility are referred for a full mental health evaluation at a facility. After the mental health evaluation, referrals are made for psychotherapy, psychoeducation, medication management, or other follow-up provided by a collaborating team of providers and as determined by the appropriate ZL care pathway (Figure 1).

When introducing mental health services at ZL and in response to limited internet infrastructure in rural Haiti,^18^ a paper-based data collection system was developed to aggregate mental health service volume at ZL health facilities each month. At each facility, the psychologist would manually count the total number of mental health patient visits that occurred that month. Practically, there were several implementation challenges with this system, including significant labor required to manually count patient visits each month, which led to long delays in submitting data. Variability in how counts were completed across facilities and having only aggregate data available—meaning it was not possible to count or monitor individual patients—reduced the validity and usability of data. It was also not possible to include the community-level activities of community health workers using this manual approach. This context and the lack of electronic HMIS for mental health in low-resource settings globally informed the programmatic decision in late 2015 to develop and implement a digital data collection system for mental health data at ZL facilities. The goal was to develop a system that could longitudinally and confidentially collect patient data to enable data-driven programmatic decision making for mental health services and would be effective in rural Haiti. The project was intended to document only facility-level activities, with the goal that community-level community health worker data would be added later.

At the time of introducing mental health services at ZL and in response to limited internet infrastructure in rural Haiti, a paper-based data collection system was developed to aggregate mental health service volume at ZL facilities each month.

## PROGRAM INTERVENTION

To design the digital data collection system, the project team, comprised of ZL staff and PIH staff, conducted a series of in-depth individual meetings over about 1 month with the following stakeholders: ZL leadership; the ZL information technology team; ZL monitoring and evaluation team; members of the ZL and PIH mental health teams; health facility medical directors and providers; and the PIH medical informatics team. In addition to aiming to increase the buy-in of each of these stakeholders, these meetings collected input on the appropriate and feasible platform, content, technical specifications, and staffing for the data collection system.

### Design

**FIGURE 2:**
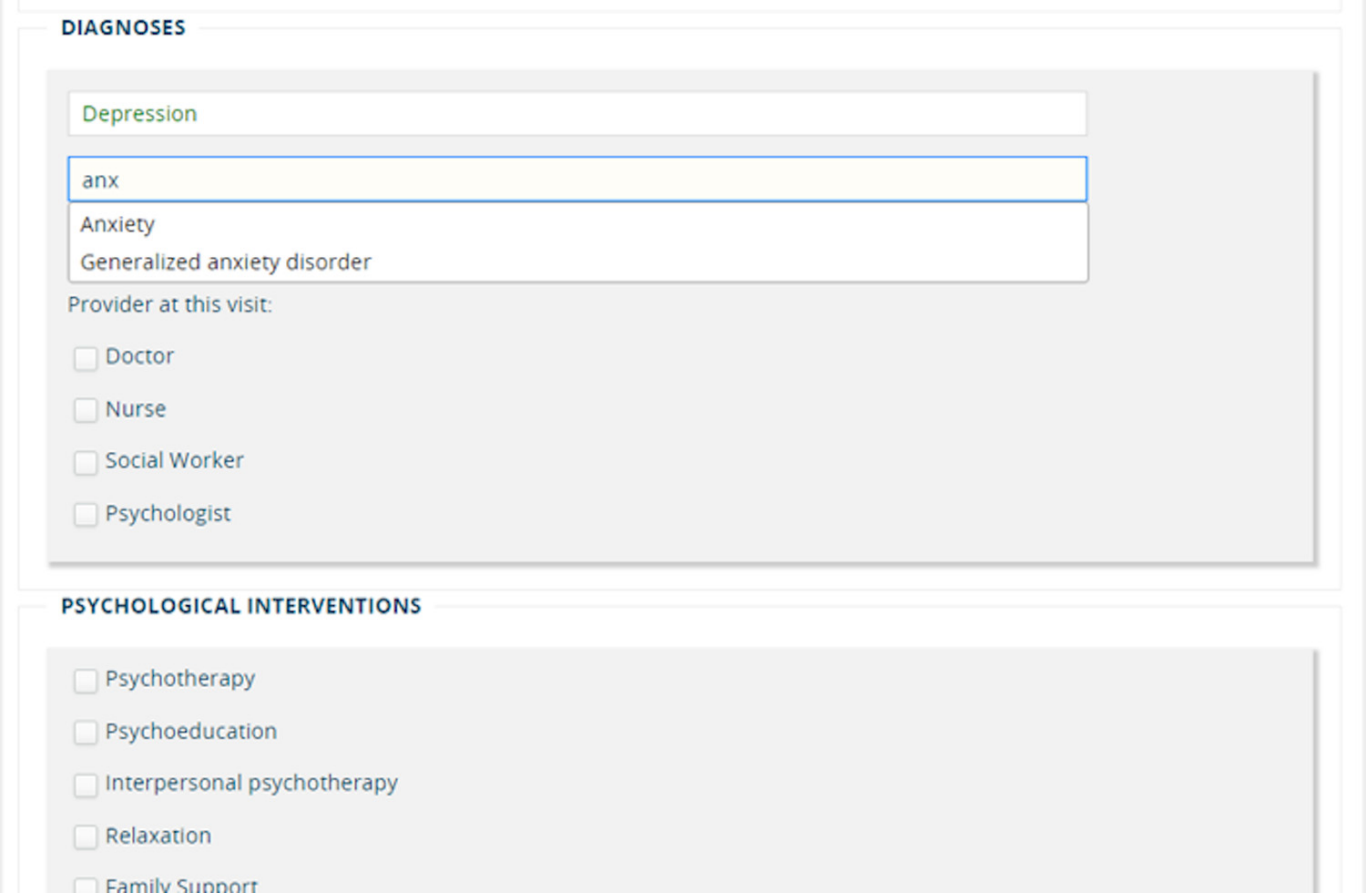
Diagnoses and Psychological Interventions Sections of English Version of Digital Data Collection Form With Comorbid Depression and Anxiety Example Used in Rural Haiti

Based on input from the individual meetings, the Open Medical Record System (OpenMRS), an open-source medical record system, was chosen for system development. Though this was the first known use of OpenMRS for mental health data, it was recommended because it was already in use elsewhere within the ZL system, allowed different permissions for different users, and is widely used in LMICs for electronic data collection.^19^^–^^21^ Informed by indicators used more broadly at ZL, along with best practices for mental health data collection in LMICs, 87 administrative (e.g., type of provider present at the visit), clinical (e.g., diagnoses), and service provision (e.g., interventions provided) indicators were identified that would need to be derived from the data collection system (Figure 2). ^22^ A data collection form was developed to be able to collect the data elements needed to calculate these indicators. Draft data collection forms were initially developed in Microsoft Word, which allowed an iterative process of refining wording, order, and content by the PIH and ZL mental health teams. Based on best practices in HMIS and feedback from the PIH medical informatics team, structured data entry was used wherever possible to record the data elements, in the form of dropdowns or auto-complete fields to ensure ease of use for users and reduce skipped fields.^21^^,^^23^

Since the system was intended to facilitate programmatic and clinical decision making, key design inputs were made from providers and clinical supervisors. Initially, the intention was to include only high-level diagnoses (e.g., depression, anxiety, or psychotic disorders) to ease diagnosis among task-shared providers who had less experience with diagnosis. However, psychologists requested that a much wider and more dimensional range of diagnoses be included within the platform (e.g., mild, moderate, or severe depression) so that they could continue to learn about the full range and severity of mental health diagnoses over time and become more specific in their clinical documentation. Clinical supervisors also supported this design decision as it would help ensure patient safety if supervisors could more easily track severity. Another key design input from clinical supervisors was the inclusion of space for free-text clinical notes alongside structured data entry. Free-text notes were not intended to be used to assess data quality or calculate indicators. However, within mental health care, free-text clinical notes are an important part of clinical documentation alongside quantitative measures and inform both safe longitudinal care of patients and clinical supervision of trainees.^24^

The digital data collection system was intended to facilitate programmatic and clinical decision making, so key design inputs were made from providers and clinical supervisors.

Based on ZL leadership, monitoring and evaluation, and mental health team feedback, it was determined that psychologists were the most appropriate cadre of staff for mental health data collection because they spent the most time working with mental health patients and learning about mental health conditions and had managed the prior paper-based mental health data visit counts. Additionally, they often took on the unofficial role of patient navigator for mental health patients at ZL health facilities and were, therefore, well placed to enter data on patient interactions with nurses and physicians that related to mental health. Clinical supervisors of psychologists suggested that data for each day's patients be entered at the end of each day to protect time for psychologists to have patient sessions earlier in the day.

Stakeholders also underscored the importance of the system being able to run with little to no internet access, as 10 of 11 health facilities did not have internet at the time of system development. At the only site that had internet, the mental health data collection system was integrated with other data systems, but at others, mental health data was siloed. The system was developed to run without internet on a series of password-protected Lenovo laptops distributed to each facility and stored in locked offices. A user ID and password were also necessary each time a person accessed the data collection system, which further ensured data privacy. Alphanumeric identifiers unique to each patient were generated by the laptops so that all data exports were anonymized and data exports did not undermine patient privacy. Minimizing the loss of collected data in a rural environment with limited infrastructure and tech support was also critical. Data backups were automated to run any time a laptop was connected to wireless internet. Data exports were coordinated to run and be submitted to the project team during monthly meetings held at a central facility, which also had internet. System performance and the need for any maintenance were monitored daily by psychologists at each site, who would alert the project team to any issues and receive tailored support, as well as monthly by project staff at the centralized meetings. It was determined that clinic-based paper forms would be retained at least during the initial rollout of the project to be able to conduct an initial quality evaluation of the digital system.

Stakeholders also underscored the importance of the system being able to run with little to no internet access.

### Implementation

After completing the design stage, pilot testing of the data collection system on a test server before deployment led to further refinement and the development of training materials and standard operating procedures for data collection (Supplement). The system was piloted for several weeks in April 2016 at 2 of 11 health facilities, leading to further minor revisions and refinement of training materials. Subsequently, a full-day training on entering data, with supervised practice, was provided to psychologists representing all ZL facilities, and the platform was rolled out to all 11 health facilities between May and July 2016. Any data not collected from January 2016 to the date of rollout at that facility was retroactively entered with the support of the ZL monitoring and evaluation team.

### Evaluation

Several strategies were used to evaluate the project 6 months after its complete rollout across the ZL system. In recognition of the fact that patients might have attended mental health services at 2 nearby sites concurrently, overlap in individuals between site caseloads was examined. To assess the reliability of data collection both overall compared to the prior paper-based system, the percentage of monthly reports successfully submitted via the digital data collection system was calculated and compared to the percentage of monthly reports submitted via the prior paper-based system.

To assess data validity, the team assessed concordance between the Zanmi Lasante Depression Symptom Inventory (ZLDSI), a measure of depression developed and validated in Haitian Creole, as documented in the digital system and original paper charts.^25^ The digital system was defined as concordant with the paper system if the value recorded in the digital system exactly matched the value in the paper original. The ZLDSI was selected as the measure of concordance because it was the clinical tool used most widely at mental health patient encounters. Concordance was assessed at 2 sites identified by clinical supervisors as being representative of the mental health patient cohort at ZL, with 1 site representing smaller facilities and 1 site representing larger facilities. All data on patients with depression digitally recorded at the 2 representative facilities between January 1, 2016, and January 31, 2017, were extracted. Of a possible 456 patients, 40 patients were randomly selected at 1 site, and 25 of a possible 255 were randomly selected from the second site. For each patient, the first and most recent visits for depression at which a ZLDSI score was recorded were selected. Two project coordinators searched for corresponding paper ZLDSI forms in facility archives, recorded whether there was a match with the digital system, and made de-identified copies of paper originals. A third project team member verified concordance with the de-identified copies.

To assess data validity, the team assessed concordance between the Zanmi Lasante Depression Symptom Inventory, a measure of depression developed and validated in Haitian Creole, as documented in the digital system and original paper charts.

To evaluate the system's ability to calculate usable indicators, 4 priority indicators were selected, 1 indicator representing a key component of care for all new patients and 1 indicator each to represent depression, epilepsy, and psychotic disorder, the key areas of mental health care provided at ZL. The 4 indicators were: ZLDSI score at first encounters for all new patients; ZLDSI score at every visit for patients with depression; documentation of number of seizures over the past 30 days at every encounter for patients with epilepsy; and a recorded score on the Abnormal Involuntary Movement Scale (AIMS) at every encounter for all patients being prescribed an antipsychotic medication. As described above, the ZLDSI is a locally developed depression screening tool and is meant to be used at first visits with all new patients and all visits for patients with depression. Seizure frequency is widely recognized as a key outcome measure in treating epilepsy, which is commonly included in mental health care in LMICs.^26^^,^^27^ The AIMS is a 12-item scale administered and scored by clinicians to detect the occurrence of dyskinesias in patients receiving neuroleptic medications.^28^ Percentages were calculated for recorded ZLDSI screens of the total number of first visits, recorded ZLDSI screens of the total number of depression visits, recorded seizure frequency of the total number of epilepsy visits, and recorded AIMS results of the total number of visits for patients on antipsychotic medication.

## RESULTS

Between January 2016 and January 2017, the digital data collection system recorded 2,445 unique mental health patients across ZL sites. Only a handful of these patients attended multiple facilities concurrently. The digital system was only missing 5 (3.5%) of 143 possible monthly reports across this period. In comparison, between January 2013 and December 2015, the paper data collection system was missing 45 (11.0%) of 409 possible monthly reports.

When examining concordance between the digital system and paper archives, of 74 forms searched for at the first sampling site, there was concordance for 55 forms (74.3%). Eighteen (24.3%) forms could not be found in the facility archives, and 1 (1.4%) form was found but did not match. For the 50 forms sampled at the second site, there was concordance between originals and the digital system for 49 forms (98.0%). One (2.0%) form was found that did not match the paper original.

Regarding the system's ability to calculate indicators, all 4 indicators could be calculated. A ZLDSI score was recorded for 1,551 (63.4%) of first encounters and 1,240 (89.7%) visits for depression. Of the encounters for patients with epilepsy, 3,000 (82.5%) documented past month seizure frequency. An AIMS score was recorded for 28 (2%) of visits for patients on antipsychotic medications. These findings led to the identification of 2 new initiatives. The lower proportion of depression screen completion at first encounter for a mental health concern, relative to depression screen completion at follow-up visits for a person with a known depressive disorder, suggested that the role of the depression screen within the ZL health system needed clarification, which was done via retraining integrated into mental health program meetings. Additionally, use of the AIMS for patients prescribed antipsychotic medication was very low, suggesting that this tool was not being used appropriately in clinical encounters and needed retraining, which was done via clinician refresher trainings on medication management of psychotic disorders.

## IMPLEMENTATION CHALLENGES AND STRATEGIES

The team encountered several implementation challenges in deploying the digital data collection system. Psychologists reported concerns that the digital system, which was substantially more detailed than the prior paper-based system, had been built to monitor their work and to assign blame if they did not perform well. Additionally, psychologists initially took an average of 17 minutes to complete a digital form for a single patient encounter. As a result, psychologists reported that they perceived that the digital data collection system would substantially increase their workload. Having retained paper records alongside the digital system to be able to evaluate the validity of the digital system further contributed to this concern, as psychologists and other providers were recording data during patient encounters on paper and then entering summary data at the end of the day digitally. One option would have been to transition to a more efficient point of care digital system, but ZL did not yet have the necessary infrastructure. Psychologists and other providers also continued to benefit from being able to use paper forms during sessions as new staff were hired due to turnover and as the scope of mental health care expanded at ZL to include new clinical skills. As a result, paper forms were retained longer than originally intended, despite the duplication.

To increase psychologists' buy-in, we took an approach consistent with both change management best practices and training providers in new clinical areas in global health.^29^^,^^30^ Project team members traveled to health facilities to help psychologists with data entry and to provide technical support for several months while providing ongoing supervision and refresher training as one would with a new clinical intervention. The project team also framed data presentations back to psychologists around the system's strengths, using digitally collected data to reward high-performing individuals at an annual ZL meeting. The emerging ability to systematically examine clinical data, such as the ZLDSI or the AIMS, further demonstrated the utility of the new data collection system to both supervisors and psychologists by enabling them to identify areas in which targeted retraining was needed (e.g., when to use the AIMS). Additional training then helped providers become clearer, more confident, and more skilled in their roles. Over several months, psychologists' speed of data entry and buy-in increased. The project team also made clear to psychologists from the beginning that this system could and would evolve and psychologists began to share suggestions for future versions. A key piece of psychologist feedback that emerged was to add decision support prompts directly into the data collection system to support in-service training and help remove the need for paper tools and the program team began to work toward this goal.

Another key challenge was created by the rapid increase in patient demand for mental health services at ZL facilities. In 2016, ZL recorded 8,100 mental health patient visits across sites, an increase from 4,696 in 2015, as services became more widely known in the area. As psychologists provide care for the majority of patients with mental health concerns at ZL, it became increasingly challenging for them to keep up with clinical duties and also manage the digital data collection system. Though psychologists' speed in entering data had improved, at the end of 2016, psychologists reported to the project team that they were experiencing difficulty keeping pace with both growing clinical caseloads and data collection. As a result, psychologists were working longer hours and monthly reports were being submitted later than scheduled. The project team became concerned that, even with removing paper forms, managing a growing clinical caseload alongside data collection would lead to psychologist burnout and undermine patient care, as well as missing data and data loss.

Another key challenge was created by the rapid increase in patient demand for mental health services at ZL facilities.

Starting in early 2017, a series of conversations were held with key stakeholders from the PIH and ZL mental health teams to begin to generate support for a shift to data clerks, identify funding for these additional staff, and develop job descriptions and training materials. Data clerks were hired in mid-2018 and deployed to each facility to take over digital data collection of mental health data from psychologists.

## LESSONS LEARNED

Despite facing implementation challenges, ZL psychologists were able to consistently and electronically collect accurate data about mental health patient encounters at ZL health facilities that could be used to calculate priority indicators that informed decision making. These findings, which are among the first for routine mental health data collection in a low-resource setting, suggest that digital data collection systems can be effective even in very low-resource settings and are consistent with the findings of other implementers.^20^^,^^31^ The challenges experienced, including that the digital data collection system increased workload for what was already a small and resource-limited staff, are consistent with those that have been reported by the few other mental health HMIS projects in LMICs.^32^^–^^34^ The implementation challenges this project faced helped inform several key lessons learned.

The challenges experienced, including that the digital data collection system increased workload for staff, are consistent with those that have been reported by the few other mental health HMIS projects in LMICs.

### Build Provider Buy-In and Capacity With Recognition and Supportive Supervision

The ongoing presence of the project team at health facilities and use of data to recognize high-performing psychologists were key strategies to building psychologist buy-in for and skill using the digital data collection system as initially deployed. Ongoing supportive supervision, while time-consuming, and opportunities for reward to users should be planned from the initial project stages to minimize challenges.

### Determine the Role of Maintaining Paper Records

Despite the greater data validity offered by a digital data collection system, systems for paper records may also need to be maintained in low-resource settings. This could be just in the short term to establish the accuracy of a new digital system, as we initially intended with this project, or possibly because there is no other way for patients to access their own records. In this project, it became clear the paper forms helped providers who were new or who were learning new interventions feel more supported. There was a training benefit to maintaining the paper forms longer than initially intended alongside the digital data collection system, just as there was a data quality benefit to introducing the digital data collection system in addition to the paper forms. In the short term, duplicate data entry was shifted to data clerks to help reduce the negative effects of this duplication on psychologists. In the longer term, the integration of decision support prompts directly into the digital data collection system offered a way to remove duplication while still maintaining support for in-service training for task-shared providers. Maintaining paper records for any period of time when introducing a digital data collection system increases workload, and the advantages and disadvantages should be carefully considered by program managers.

### Address Staffing Needs

Our experience supports the importance of implementers being realistic about the time needed for data collection and the potentially large and growing caseloads that providers may be taking on in mental health care systems in contexts with otherwise limited access to mental health care.^35^ Within the ZL system, when introducing the electronic data collection system, there was simultaneously an 83% annual increase in service demand. The decision to shift data collection responsibilities to mental health data clerks was feasible due to existing grants and was essential to prevent psychologist burnout and ensure quality data collection at the time.^36^ Such a decision may not be needed in other health systems, depending on available staffing and how quickly service demand is growing. Additionally, such a decision to add data clerks may not be feasible in other settings and may not be sustainable at a larger scale or without external funding. A key lesson for program managers regardless of setting is that it is important to identify who is responsible for collecting mental health data in a given context and ensure that this does not interfere with their other existing clinical duties and other responsibilities. Directly integrating mental health data collection within broader data collection systems that exist or are being developed—which was not possible in our context except for in 1 example where internet was available—would leverage existing resources for mental health and may help address this issue of sustainable staffing.

A key lesson is to identify who is responsible for collecting data and ensure that data collection does not interfere with their other responsibilities.

### Offset Financial Costs by Integrating With Existing Data Collection Systems

In addition to the costs of staff time to develop the digital data collection system and enter and manage data, this project also had other costs. Primarily, these were the cost of computers (US$1,300 each) as OpenMRS is free. For the relatively low cost of approximately US$15,000, it was possible to substantially improve mental health data collection. This, in turn, allowed better clinical and programmatic decision making within the ZL care system, enabled ZL to much more clearly describe its impact internally and externally, and facilitated ZL to generate more funding. However, the additional expense of the computers, while manageable at ZL due to existing grant funding, would likely not have been sustainable without external funding and would likely not scale across an entire country due to limited financial resources for mental health.^37^ We could have used lower-priced tablets or scanner-supported data collection in which paper forms are digitized to help further reduce costs and improve project sustainability and scalability.^38^^,^^39^ Digital data collection innovations developed and deployed by necessity during the coronavirus disease (COVID-19) pandemic may help guide the way in better understanding the effectiveness, feasibility, and acceptability of newer technologies for data collection within mental health care in rural LMICs. Again, integration of mental health data collection directly within other data collection systems and innovations may offer the most financially sustainable and scalable option.

## CONCLUSIONS

We have described the development, implementation, and evaluation of a digital data collection system for mental health data in rural Haiti led by psychologists. This is the first data collection system of this type for mental health data in Haiti and represents one of the few routine digital mental health data collection systems tested in an LMIC globally. The primary goal for this system was to improve data collection so that it could facilitate decision making, which was achieved. However, the need to eventually introduce data clerks to ensure both quality psychological care and data collection occurred reveals an important ongoing staffing challenge in mental health data collection in LMICs. Moreover, task-shared providers may need access to paper forms or decision support tools to structure patient interactions. Leveraging lessons learned to identify and test more sustainable and scalable models for mental health data collection that can also support in-service training for task-shared providers represents an important future area of work in global health implementation.

## Supplementary Material

GHSP-D-20-00486-supplement.pdf

## Translation

**En Français**

**Développement d'un système innovant de collecte de données numériques dans la prestation de soins routiniers de santé mentale en région rurale haïtienne**

Introduction. Les systèmes de gestion de l'information sanitaire (SGIS) sont reconnus efficaces quand ils étayent la validité des données de santé permettant ainsi aux équipes soignantes de prendre des décisions programmatiques et des résolutions nationales au regard des objectifs internationaux de développement. En 2015, la santé mentale a été incluse dans les Objectifs de Développement Durable (ODD), pourtant il existe peu d'applications de SGIS de quelque type que ce soit dans la pratique des soins de santé mentale dans les milieux à ressources limitées. Zanmi Lasante (ZL), l'un des plus grands fournisseurs de soins de santé mentale en Haïti, a développé un système de collecte de données numériques pour la santé mentale à travers 11 établissements sanitaires ruraux. Intervention du programme. Nous décrivons le développement, la mise en œuvre et l'évaluation du système numérique de collecte de données de santé mentale à ZL. Pour évaluer la fiabilité du système, nous avons déterminé le nombre de rapports mensuels manquants. Pour évaluer la validité des données, nous avons calculé la concordance entre le système numérique et les dossiers papier dans deux établissements. Pour évaluer la capacité du système à éclairer la prise de décision, nous avons spécifié et calculé quatre indicateurs prioritaires. Résultats. Il manquait au système numérique 5 des 143 rapports mensuels dans tous les établissements et la concordance avec les dossiers papier était de 74,3% (55/74) et 98% (49/50). Il a été possible de calculer les quatre indicateurs, ce qui a entraîné des changements programmatiques dans deux cas. En réponse aux défis de la mise en œuvre, il a été nécessaire d'utiliser des stratégies pour stimuler la participation des prestataires dans un premier temps et finalement d'introduire des officiers de données dédiés à la saisie régulière des données permettant ainsi aux prestataires de consacrer plus de temps aux activités cliniques. Leçons apprises. Tout en démontrant le potentiel de la collecte de données numériques sur la santé mentale dans un milieu rural à faibles ressources, nous avons constaté qu'il était nécessaire de prendre en compte le rôle continu que joue parallèlement les dossiers papier. Nous avons également identifié le défi d'établir le juste équilibre entre les responsabilités cliniques et celles correspondantes à la saisie des données compte tenu du nombre restreint de personnel. Des efforts réguliers sont nécessaires afin de développer des modèles réellement durables et évolutifs pour la collecte de données sur la santé mentale dans les milieux à ressources limitées.

**Principales conclusions:**

- L'équilibre entre le temps consacré aux tâches cliniques et le celui alloué à la saisie des données a été un défi majeur quand les prestataires de soins y sont impliqués.

- L'apport de commentaires sur les données à l'intention des utilisateurs, la supervision continue et les primes de reconnaissance aux acteurs performants peuvent contribuer à augmenter l'adhésion aux nouveaux systèmes de collecte de données.

- Le maintien des supports papier en plus des systèmes de collecte de données numériques ou l'inclusion d'outils d'aide à la décision directement dans les systèmes de collecte de données numériques peut être nécessaire pour soutenir pleinement l'apprentissage des prestataires dans un système de partage de tâches au sein du personnel de santé mental.

**Principaux enseignements:**

- Les gestionnaires de programme doivent soigneusement considérer une dotation durable en personnel dès la conception des projets de collecte de données numériques.

- Les gestionnaires de programme doivent envisager comment les systèmes de collecte de données numériques peuvent intégrer des outils d'aide à la décision pour soutenir l'apprentissage continu des prestataires partageant des tâches communes.

- Les décideurs devraient intégrer la collecte de données de santé mentale dans d'autres systèmes de données afin de s'assurer de l'infrastructure et des ressources en faveur de la santé mentale.

**Peer Reviewed**

**First published online:** November 30, 2021.
