# Supplementary material for: Development of an Innovative Digital Data Collection System for Routine Mental Health Care Delivery in Rural Haiti
Source: Glob Health Sci Pract. 2021 Dec 31;9(4):990–9. doi: 10.9745/GHSP-D-20-00486 (PMC8691881; doi:10.9745/GHSP-D-20-00486)
Supplement: GHSP-D-20-00486-supplement.pdf [file GHSP-D-20-00486-supplement.pdf]

Zanmi Lasante

Supplement to: Rose AL, Fenelon DL, Fils-Aimé JR, et al. Development of an innovative digital data collection system for routine mental health care delivery in rural Haiti. *Glob Health Sci Pract*. 2021;9(4). https://doi.org/10.9745/GHSP-D-20-00486

Supplement: English version of standard operating procedures developed in April 2016 to train psychologists in use of digital data collection system for mental health data

Zanmi Lasante **Supplement to**: Rose AL, Fenelon DL, Fils-Aimé JR, et al. Development of an innovative digital data collection system for routine mental health care delivery in rural Haiti. *Glob Health Sci Pract*. 2021;9(4). https://doi.org/10.9745/GHSP-D-20-00486

How to login into the Mental Health Database:

For XXX:

1. Make sure your computer is connected to the internet
2. Go to this link: xxxxxxxxxxxxxx
3. Enter your username and password and make sure xxxxxxxxxxxxxx is selected
4. Click on connect.

For XXX :

1. Click on the OpenMRS logo on your desktop. The picture is the one with PIH hands.
2. Enter your username and password and make sure your health facility is selected.
3. Click on connect.
4. NOTE: You do not need an internet connection to use this database!

Supplement to: Rose AL, Fenelon DL, Fils-Aimé JR, et al. Development of an innovative digital data collection system for routine mental health care delivery in rural Haiti. *Glob Health Sci Pract*. 2021;9(4). https://doi.org/10.9745/GHSP-D-20-00486

How to enter data in the Mental Health Database:

For XXX:

1. When you login, you will be on the home page.
2. Registering a patient:
   a. Patients at xxxxxxxxxxxxxx should already be registered, so you should not need to register them.
3. Starting a visit:
   a. You can start a visit by searching for them by name on the top of the home page. This will pull up a list of patient's with that name.
   b. When you find their name, click on it and it will bring you to their personal dashboard. If you end up in the wrong patient's dashboard you can always start over by clicking on the Zanmi Lasante logo on the top left corner.
   c. To start a new mental health visit from their personal dashboard visit click on Sante Mental in the blue box on the right side. This will bring you to the Mental Health encounter form.
4. Completing the Mental Health Encounter Form:
   a. Community Referral
      i. Was this patient referred to the health center from the community? Check or write in anyone who referred them or leave it blank if it is not relevant.
   b. Scores

i. What were the clinical scores for this patient and this encounter? You should enter their result for:
   - ZLDSI
   - CES-D (if they a teenager)
   - CGI (note this is broken out into three different scores, like on the paper form)
   - WHODAS (this one is required. You will not be able to complete the form without entering a WHO-DAS score)
     a. After entering a score for the WHO-DAS, enter a number for each of the three prompts about lost productivity
     b. If you have not conducted a WHO-DAS with the patient because they were screened with it less than thirty days ago, please enter a 0 for the WHO-DAS score.
   - AIMS

ii. Did the patient have any seizures this month? If yes, enter that information. If the patient did not have any seizures this month, enter a zero.

iii. Did you conduct a suicidal evaluation of this patient? Indicate whether or not you did.

iv. Check the box if the patient has suicidal ideation from your assessment.

c. Diagnoses:

i. There are three boxes here. Type in up to three diagnoses for the patient at this encounter. When you

start typing in the word the options will appear on the screen in French and you can click on them. You can also enter each individual field.

ii. Did the patient seen a doctor, a nurse, a psychologist, or a social worker at this visit? Check all that apply

d. Psychological interventions:

i. Check all of the interventions that you conducted with the patient or that the patient is receiving.

e. Medications:

i. Check all of the medications that the patient is taking.

ii. In the additional comment section, please write any information about dosage or side effects. You can also use this space to record any information other non-mental health medication the patient may be taking.

f. Plan:

i. Does this patient have a security plan? If so, check the box.

ii. Was the patient hospitalized at this visit? If so, check the box.

iii. Was the patient referred to a doctor, a nurse, a social worker, or a CHW? If so, check the box that applies.

iv. Please write any additional notes on the patient in the comment box.

- <u>This is where you should write in any information on medication dosage, other medications, etc…</u>

v. If the patient has a follow-up visit scheduled enter that in the return visit date box.

Supplement to: Rose AL, Fenelon DL, Fils-Aimé JR, et al. Development of an innovative digital data collection system for routine mental health care delivery in rural Haiti. *Glob Health Sci Pract*. 2021;9(4). https://doi.org/10.9745/GHSP-D-20-00486

g. Press submit or else the form will not be saved!

For XXX :

1. When you login, you will be on the home page.
2. Registering a patient:
   a. Since we are rolling out a new database, patients will need to be registered. Once a patient has been registered in the database you will just be able to search for their name at future visits and you do not need to reregister then.
   b. Click on Register a patient.
   c. Type in their name and click the box that says register a patient again. This will bring you to the registration page.
   d. NOTE: FOR ANY FIELD THAT IS REQUIRED BUT YOU DO NOT KNOW THE ANSWER, WRITE UNKNOWN.
   e. If the patient was registered on the day you are entering this data leave the box “Today” checked. If the patient was registered on a different day from the day you are entering the data, uncheck the box and enter the date they were registered.
   f. Click on Name and enter their name. First name and last name are required.
   g. Click on gender and select male or female. Choosing one is required.
   h. Click on birthdate. Either their exact birth date or an estimated age is required.

i. Click on mother's first name. If they do not know this, this is not required.

j. Click on address and enter in their address. This is required. Instead of entering in pays, department, commune, section communale, and localite habitation individually, you can also complete this by searching for their localite in the box at the top where it says "Recherche rapide par Localité Habitation."
   i. If you do not know the patients address, please enter the address of the health center instead.
   ii. If you cannot find the patients localite habitation from the list of options, please leave it blank.
   iii. Please use the box at the bottom where it says "Adresse" to write their street name or any descriptive information about their house.
   iv. If the patient is from outside Haiti, select Yon Lot Pays from the Pays field. You will then need to enter a dash (-) for department, commune, and section communale.

k. Click on phone number. If they do not have a phone, this is optional.

l. Click on Place of birth and enter their place of birth. This is required. You can also complete this by searching for their location in the box at the top where it says "Recherche rapide par Localité Habitation"

m. Click on civil status and enter their civil status. This is optional.

n. Click on occupation and enter their occupation. This is optional.
o. Click on religion and enter their religion. This is optional.
p. Click on contact name and enter a contact for this person. This is required.
q. Click on contact address and enter their contact's address. This is required.
r. Click on contact phone number and enter their contact's phone number. This is required.
s. Click on confirm. The information will be lost if you do not click Confirm!
t. Once a patient has been registered once you will not have to do it again, but you can go back later and edit this information if you need to.

3. Starting a visit:
   a. To start a visit, first you need to search for and click on the patient's name. This will bring up a list of patients with that name.
   b. When you find their name, click on it and it will bring you to their personal dashboard. If you end up in the wrong patient's dashboard, you can always start over by clicking on the Zanmi Lasante logo in the top left corner.
      i. When you are in the personal dashboard, you can add in a patient's paper dossier number if you would like. This can be helpful if you want to go back and check a patients paper files later based on the database.

Zanmi Lasante **Supplement to**: Rose AL, Fenelon DL, Fils-Aimé JR, et al. Development of an innovative digital data collection system for routine mental health care delivery in rural Haiti. *Glob Health Sci Pract*. 2021;9(4). https://doi.org/10.9745/GHSP-D-20-00486

1. You can do this by clicking on "Add" next to Reference number in the top right corner and typing in a number and then clicking confirm.

c. To start a visit from their personal dashboard, click on Clinical dashboard.

d. Click on Start visit. This will bring you to the mental health form.

e. Click on the pencil on the right side of the page.

4. <u>Completing the Mental Health Encounter Form:</u>

   a. Community Referral

      i. Was this patient referred to the health center from the community? Check or write in anyone who referred them or leave it blank if it is not relevant. In the other box, you can write in the name of a CHW.

   b. Scores

      i. What were the clinical scores for this patient and this encounter? You should enter their result for:

         1. ZLDSI
         2. CES-D (if they a teenager)
         3. CGI (note this is broken out into three different scores, like on the paper form)
         4. WHODAS (this one is required. You will not be able to complete the form without entering a WHO-DAS score)

            a. After entering a score for the WHO-DAS, enter a number for each of the three prompts about lost productivity.

b. If you have not conducted a WHO-DAS with the patient because they were screened with it less than thirty days ago, please enter a 0 for the WHO-DAS score.

5. AIMS

ii. Did the patient have any seizures this month? If yes, enter that information. If the patient did not have any seizures this month, enter a zero.

iii. Did you conduct a suicidal evaluation of this patient? Indicate whether or not you did.

iv. Check the box if the patient has suicidal ideation from your assessment.

c. Diagnoses:

i. There are three boxes here. Type in up to three diagnoses for the patient at this encounter. When you start typing in the word the options will appear on the screen in French and you can click on them. You can also enter each individual field.

ii. Did the patient seen a doctor, a nurse, a psychologist, or a social worker at this visit? Check all that apply

d. Psychological interventions:

i. Check all of the interventions that you conducted with the patient or that the patient is receiving.

e. Medications:

i. Check all of the medications that the patient is taking.

ii. In the additional comment section, please write any information about dosage or side effects. You can also use this space to record any information other non-mental health medication the patient may be taking.

f. Plan:

i. Does this patient have a security plan? If so, check the box.

ii. Was the patient hospitalized at this visit? If so, check the box.

iii. Was the patient referred to a doctor, a nurse, a social worker, or a CHW? If so, check the box that applies.

iv. Please write any additional notes on the patient in the comment box.

1. This is where you should write in any information on medication dosage, other medications, etc…

v. If the patient has a follow-up visit scheduled enter that in the return visit date box.

g. Press submit or else the form will not be saved!

Supplement to: Rose AL, Fenelon DL, Fils-Aimé JR, et al. Development of an innovative digital data collection system for routine mental health care delivery in rural Haiti. *Glob Health Sci Pract*. 2021;9(4). https://doi.org/10.9745/GHSP-D-20-00486

Additional Notes, Laptop Safety, Backups, and Running Reports:

1. Data should be entered at the end of every day for the patients seen that day. If you need to enter the data on a different day from when you saw the patient, **please make sure the day of the actual patient visit**, and not the day that you enter the data, is what is entered into the database.
2. The laptop holds patient level information and must be kept in a safe place. Its security is very important.
3. **The laptop must be brought with you to the monthly psychosocial meetings at** xxxxxxxxxxxxxx**. This is a critical opportunity to backup data via the internet and to run a report for MEQ.**
4. Backing up data:
   a. When you bring your computer to xxxxxxxxxxxxxx for the psychosocial meeting, you will need to open it and make sure it is connected to the internet for it to back up the data on your computer. If you do not connect to the internet the data cannot backup!
   b. You should do this at the beginning of the meeting because it may take a little while for the backup to complete.
   c. XXX will check that everyone has done this. It is very important that everyone backs up their data in case something happens to the laptops.
5. Running reports:

Zanmi Lasante **Supplement to**: Rose AL, Fenelon DL, Fils-Aimé JR, et al. Development of an innovative digital data collection system for routine mental health care delivery in rural Haiti. *Glob Health Sci Pract*. 2021;9(4). https://doi.org/10.9745/GHSP-D-20-00486

a. XXX and the MEQ team will help with running reports. This will now be a scheduled part of the psychosocial meeting.
b. To access the reports, go back to the home page by clicking the ZL logo in the top left corner.
c. Click on Reports.
d. Click on mental health data
e. Enter the start date and the end date you want the report to run for.
    i. The start date should begin at the end of the last report you ran.
    ii. The end date should be the present day.
f. Click on run.
g. The report will appear in the box that says available for download.
h. Click on download
i. Open the file in Excel by clicking on it at the bottom of your browser.
j. Save the file with your location and dates in the title:
    i. Example: XXXXMarch1-March30
k. Give this file on a flash or via email to the MEQ team. It is important that you give them a copy of this report or else the data may be lost if anything happens to your laptop.

6. Support:
    a. If you are having any problems with using the database please contact XXXX or XXX. They will be able to help or connect you to someone at XXXX who can help.
